# Unveiling the Dependence of Glass Transitions on Mixing Thermodynamics in Miscible Systems

**DOI:** 10.1038/srep08500

**Published:** 2015-02-17

**Authors:** Wenkang Tu, Yunxi Wang, Xin Li, Peng Zhang, Yongjun Tian, Shaohua Jin, Li-Min Wang

**Affiliations:** 1 State Key Lab of Metastable Materials Science and Technology, and College of Materials Science and Engineering, Yanshan University, Qinhuangdao, Hebei, 066004 China; 2 School of Materials Science and Engineering, Beijing Institute of Technology, Beijing, 100081 China

## Abstract

The dependence of the glass transition in mixtures on mixing thermodynamics is examined by focusing on enthalpy of mixing, Δ*H_mix_* with the change in sign (positive vs. negative) and magnitude (small vs. large). The effects of positive and negative Δ*H_mix_* are demonstrated based on two isomeric systems of *o*- vs. *m*- methoxymethylbenzene (MMB) and *o*- vs. *m*- dibromobenzene (DBB) with comparably small absolute Δ*H_mix_*. Two opposite composition dependences of the glass transition temperature, *T_g_*, are observed with the MMB mixtures showing a distinct negative deviation from the ideal mixing rule and the DBB mixtures having a marginally positive deviation. The system of 1, 2- propanediamine (12PDA) vs. propylene glycol (PG) with large and negative Δ*H_mix_* is compared with the systems of small Δ*H_mix_*, and a considerably positive *T_g_* shift is seen. Models involving the properties of pure components such as *T_g_*, glass transition heat capacity increment, Δ*C_p_*, and density, *ρ*, do not interpret the observed *T_g_* shifts in the systems. In contrast, a linear correlation is revealed between Δ*H_mix_* and maximum *T_g_* shifts.

Glass transition is a typical liquid-solid non-equilibrium transition occurred in all kinds of materials, however, the physics of the phenomenon remains unsolved and debated^1^,^2^,^3^. The glass transition behaviors are found to be quite sensitive to even subtle modification in molecular structure and intermolecular interactions^4^. Binary glass forming mixtures are simple and appealing systems to investigate the glass transition due to the ease in modifying the intermolecular interactions. Miscible mixtures usually show a single glass transition temperature, *T_g_*^5^, which might not necessarily be the mean value of the pure components' *T_g_*s produced by the ideal mixing rule, but is related to the mixture composition (e.g. mole fraction, *x*)^6^,^7^.

The composition dependence of *T_g_* in mixtures has been extensively studied in a number of systems, and diverse observations in the *T_g_*-*x* relationships have been reported, showing positive, negative and zero deviations from the ideal mixing rule^5^,^6^,^8^,^9^,^10^,^11^,^12^,^13^. Several models are proposed in search of the explanation for such deviations^14^, such as the most widely used Couchman - Karasz (CK) and Gordon - Taylor (GT) models^15^,^16^. The CK model takes the glass transition as an Ehrenfest second order transition, and assumes the changes upon mixing in enthalpy (Δ*H_mix_*), entropy (Δ*S_mix_*), and volume (Δ*V_mix_*) for both liquid and glass to be negligible at *T_g_*. Moreover, the glass transition heat capacity increment, Δ*C_p_*, is assumed to be temperature-independent. The GT model is proposed based on ideal volume of mixing and the linear change in volume with temperature. The two models have the similar expression as, *T_g_* = (*x*_1_*T_g_*_1_ + *k_i_x*_2_*T_g_*_2_)/(*x*_1_ + *k_i_x*_2_), where *k_CK_* = Δ*C_p_*_1_/Δ*C_p_*_2_ is determined for the CK model while for the GT model *k_GT_* = *ρ*_2_*T_g_*_2_/*ρ*_1_*T_g_*_1_, and *x_i_* and *ρ_i_* (*i* = 1, 2) denote the mole fraction and density of pure components^17^. Obviously, the two models emphasize the significance of two components' properties including *T_g_*, Δ*C_p_*, and *ρ*. Although the two models have successfully explained some experimental measurements^18^,^19^, there are a number of systems that show pronounced discrepancy between experimental measurements and the theoretical calculation made by the models^6^,^13^,^20^. Alternatively, mixing thermodynamics is argued to determine the glass transition behaviors in mixtures, and studies found that positive enthalpy of mixing generally corresponds to negative *T_g_* deviation^5^, and even a value of Δ*H_mix_* as low as 200 J/mol can result in a remarkably negative *T_g_* shift^21^. However, the evaluation of the weighted contributions to the *T_g_* shifts from both mixing thermodynamics and the properties of pure components have not been available. Moreover, systematic studies of the dependence of *T_g_* on mixing thermodynamics have not been done. Therefore, the studies of the impact of Δ*H_mix_* on the *T_g_* shifts in mixtures by changing both magnitude (small vs. large) and sign (positive vs. negative) would help gain insight into the glass transition behaviors in mixtures. Unfortunately, such studies are not accessible, in particular, the effect of small Δ*H_mix_*.

In this work, we studied the glass transitions in binary glass forming molecular systems, and systematic comparison is shown with typical scenarios of small vs. large and positive vs. negative Δ*H_mix_*. A global picture of the Δ*H_mix_* effect on the *T_g_* shifts in mixtures is presented. The results are expected to benefit the understanding of the glass transition as well as the precise evaluation of *T_g_* in poor glass formers.

## Results

**Figure 1:**
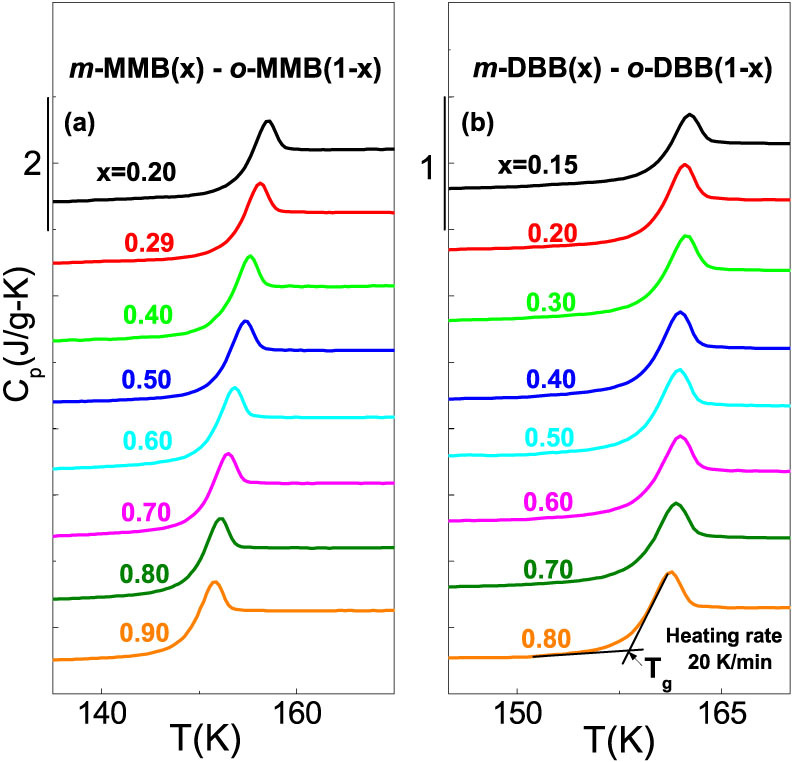
Glass transition heat capacity *C_p_* curves of two isomeric binary systems for the glass forming compositions. (a) *o*- vs. *m*- methoxymethylbenzene (MMB); (b) *o*- vs. *m*- dibromobenzene (DBB). The curves are determined at a heating rate of 20 K·min^−1^.

**Figure 2:**
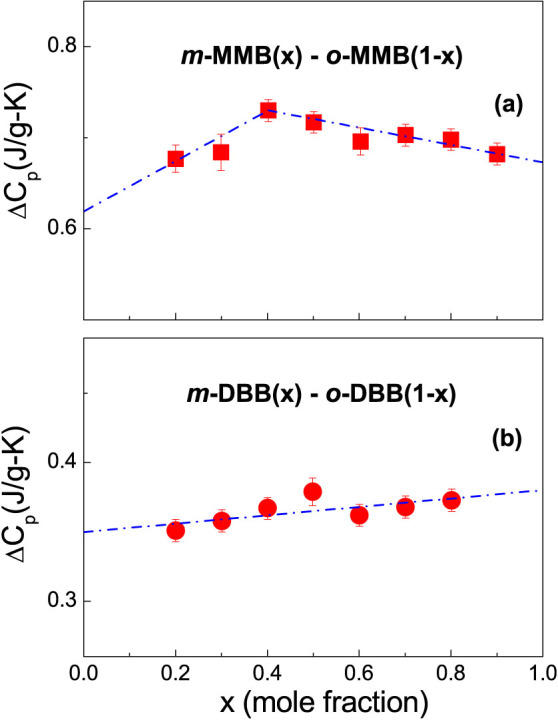
Composition dependence of the glass transition heat capacity increases, Δ*C_p_*, in two isomeric mixtures. (a) *o*- vs. *m*- methoxymethylbenzene (MMB); (b) *o*- vs. *m*- dibromobenzene (DBB). The extrapolations for the mixtures' heat capacity increment, Δ*C_p_*, are given by the blue dash-dot lines.

**Figure 3:**
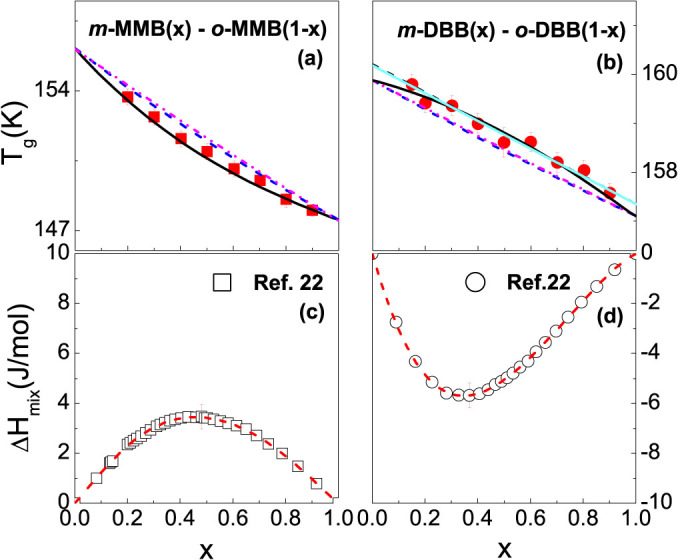
Composition dependence of *T_g_* and Δ*H_mix_* in the isomeric mixtures of *o*- vs. *m*- methoxymethylbenzene (MMB) and *o*- vs. *m*- dibromobenzene (DBB). In the upper frames (a) and (b), the red solid squares and circles are experimental values. The fitting line for the *o*- vs. *m*- methoxymethylbenzene (MMB) is given by the black solid line, while the fitting line for the *o*- vs. *m*- dibromobenzene (DBB) is expressed by the black or the light cyan solid line. The predicted values by the CK and GT models are shown by the blue dotted (CK) and magenta dash-dot-dot (GT) lines. The lower frames (c) and (d) show Δ*H_mix_* values for the two isomeric systems recorded in Ref. 22. The horizontal axes show the mole fraction of *m*- MMB (*x*) and DBB (*x*).

**Figure 4:**
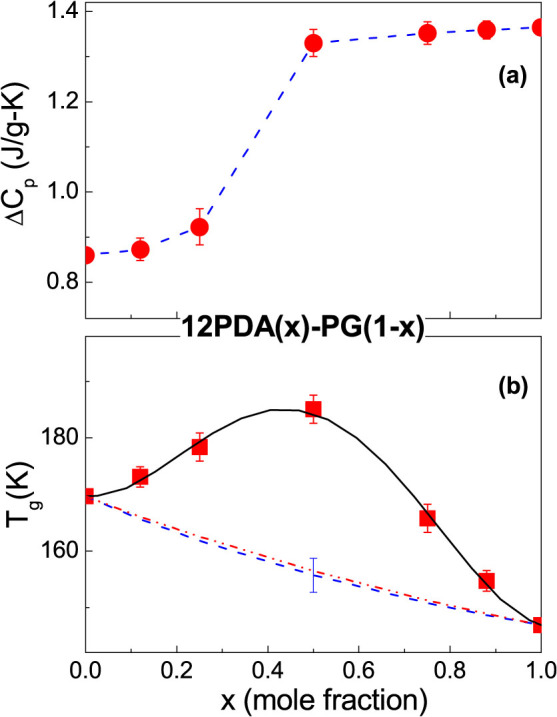
Composition dependence of the heat capacity increment, Δ*C_p_* (a), and *T_g_* (b) of 1, 2- propanediamine (12PDA) vs. propylene glycol (PG). The predicted values by the CK and GT models are shown by the blue dotted (CK) and magenta dash-dot-dot (GT) lines in frame (b).

Fig. 1 presents the *C_p_* curves around *T_g_* for the glass forming *o*- vs. *m-* MMB and *o*- vs. *m*- DBB systems. The composition dependence of Δ*C_p_* obtained from all the *C_p_* curves are plotted in Fig. 2(a) and (b) for the two systems. The Δ*C_p_* values of the pure components are estimated by extrapolating the mixtures' Δ*C_p_*, and 0.619, 0.673, 0.35 and 0.38 J/(g–K) are obtained for *o*-MMB, *m*-MMB, *o*-DBB and *m*-DBB. The composition dependences of *T_g_* are plotted in Fig. 3(a) and (b). A markedly negative *T_g_* deviation is immediately visible for the mixtures of *o*- vs. *m*- MMB, although the deviation is small. In contrast, the evaluation of the *T_g_* shift for the system of *o*- vs. *m*-DBB in Fig. 3(b) is of a bit difficulty. The fitting with marginally positive deviation or a linear fitting seems to be able to explain the experimental points, as shown by the black solid line and the light cyan solid line. The bottom line for *o*- vs. *m*-DBB is that the deviation is, by no means, negative. The pure components' *T_g_*s can be evaluated by the numeric extrapolation in Fig. 3, yielding 156.1, 147.5, 160 and 157.2 K for *o*-MMB, *m*-MMB, *o*-DBB and *m*-DBB. The Δ*H_mix_* values are also presented in Fig. 3(c) and (d), reported for the mixtures of *o*- vs. *m*- MMB and *o*- vs. *m*- DBB, with Δ*H_mix_* maxima to be 3.42 and −4.98 J/mol, respectively^22^. Fig. 4(a) and (b) show the composition dependences of Δ*C_p_* and *T_g_* for the system of 12PDA vs. PG over the whole composition, where Δ*H_mix_* maximum of 12PDA vs. PG is recorded to be −3876 J/mol^23^. A remarkably positive *T_g_* deviation is seen in the system. The Δ*C_p_* values for 12PDA and PG are determined to be 1.36 and 0.86 J/(g–K) while the *T_g_*s to be 146.8 and 169.7 K.

The explanations of the *T_g_* values in the mixtures using the CK and GT equations with the extrapolated *T_g_* and Δ*C_p_* values of the pure components are presented by the blue dotted (CK) and magenta dash-dot-dot (GT) lines in Fig. 3(a), (b) and Fig. 4(b). It appears that the calculated *T_g_* curves in terms of the CK and GT models do not satisfactorily explain the experimental measurements. Similar observations have been reported in early studies of a number of glass forming mixtures^13^,^20^. The large discrepancy suggests that, notwithstanding the importance of the properties (*T_g_*, Δ*C_p_* and *ρ*) of the pure components, the exact explanation of the *T_g_* - *x* relationship for most cases asks for more considerations.

**Figure 5:**
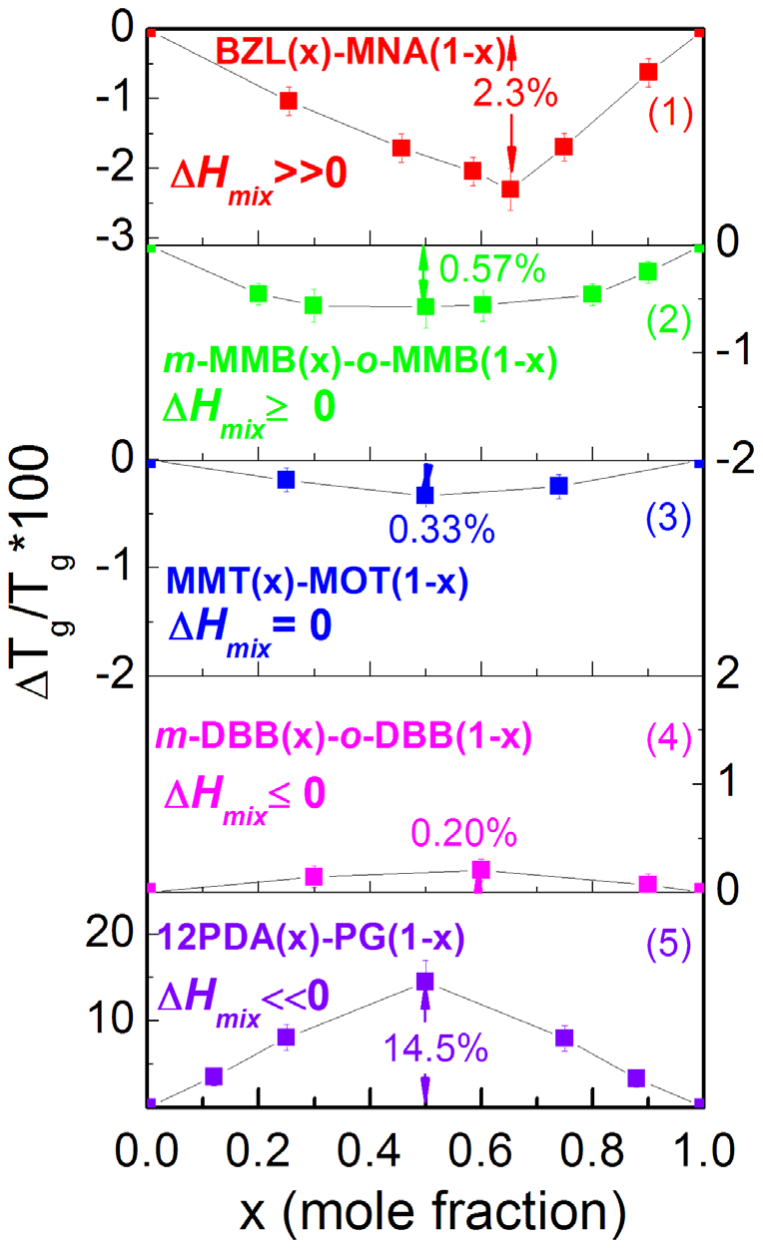
Relative *T_g_* differences, Δ*T_g_*/*T_g_*, for five typical glass forming systems, representing various mixing modes. (1) Δ*H_mix_* ≫ 0 (benzil (BZL) vs. *m*- nitroaniline (MNA)), (2) Δ*H_mix_* ≥ 0 (*o*- vs. *m*- methoxymethylbenzene (MMB)), (3) Δ*H_mix_* = 0 (methyl *m*-toluate (MMT) vs. methyl *o*-toluate (MOT)), (4) Δ*H_mix_* ≤ 0 (*o*- vs. *m*- dibromobenzene (DBB)) and (5) Δ*H_mix_* ≪ 0 (1, 2- propanediamine (12PDA) vs. propylene glycol (PG)).

The relative *T_g_* shift, Δ*T_g_*/*T_g_*, for the systems of *o*- vs. *m*- MMB, *o*- vs. *m*- DBB, and 12PDA vs. PG are shown in Fig. 5, where Δ*T_g_* is the *T_g_* difference between the measured values and the ones calculated in terms of the linear average of the pure components' *T_g_*s. Two more typical systems recorded in our recent work, benzil (BZL) vs. *m*-nitroaniline (MNA)^21^ and methyl *m*-toluate (MMT) vs. methyl *o*-toluate (MOT)^24^, are also included in Fig. 5 to represent the mixing scenarios of larger positive Δ*H_mix_* and ideal mixing, respectively. It is seen in Fig. 5 that the five systems differ greatly in the *T_g_* deviations from the ideal mixing rule. The system of 12PDA vs. PG has a remarkably large and positive *T_g_* deviation with the maximum Δ*T_g_*/*T_g_* being as high as 14.5%. In contrast, the system of BZL vs. MNA has a large and negative *T_g_* deviation with the maximum of Δ*T_g_*/*T_g_* of −2.3%^21^. For systems with smaller positive or negative Δ*H_mix_*, Δ*T_g_*/*T_g_* becomes smaller.

## Discussion

The mixing thermodynamics for the BZL vs. MNA system (Fig. 5) is recently measured showing the Δ*H_mix_* maximum of ~200 J/mol^21^, while the MMT vs. MOT system is regarded as an ideal mixing case with Δ*H_mix_* = 0^24^,^25^. Accordingly, the five cases in Fig. 5 represent the typical model systems of Δ*H_mix_* ≫ 0 (BZL vs. MNA), Δ*H_mix_* ≥ 0 (*o*- vs. *m*- MMB) Δ*H_mix_* = 0 (MMT vs. MOT), Δ*H_mix_* ≤ 0 (*o*- vs. *m*- DBB) and Δ*H_mix_* ≪ 0 (12PDA vs. PG). It is therefore evident that systems with large absolute values of Δ*H_mix_*, either positive or negative, show remarkable negative or positive *T_g_* shifts. Similar observations have been recorded in earlier experimental measurements. For example, the binary mixtures constituted by monoalcohols and amines with large and negative Δ*H_mix_*^26^,^27^,^28^ generally show large and positive *T_g_* shifts^29^. Likewise, large and negative Δ*H_mix_* is also detected in the mixtures composed of water and alkoxy alcohols, such as the aqueous systems of 2-methoxyethanol^30^ and 2-ethoxyethanol^31^, and large and positive *T_g_* deviations are observed in such mixtures^10^.

**Figure 6:**
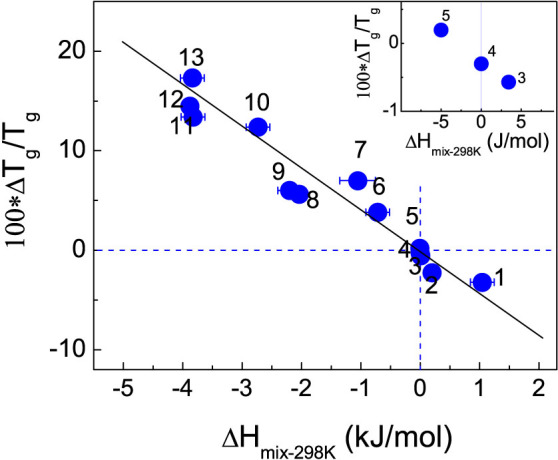
Correlation between Δ*H_mix_* and Δ*T_g_*/*T_g_* based on 13 binary molecular glass forming systems. (1) 1- butanol vs. 1- bromobutane^41^,^43^,^44^; (2) benzil (BZL) vs. *m*- nitroaniline (MNA); (3) *o*- vs. *m*- methoxymethylbenzene (MMB); (4) methyl *m*-toluate (MMT) vs. methyl *o*-toluate (MOT); (5) *o*- vs. *m*- dibromobenzene (DBB); (6) chloroform (CFM) vs. toluene^8^,^34^; (7) diethylether (DEE) vs. dichloromethane (DCM)^8^,^37^; (8) methanol vs. triethylamine^29^,^33^ (9) 2-ethyl-1-hexanol (2E1H) vs. 2-ethyl-1-hexylamine (2EHA)^25^ (10) chloroform (CFM) vs. diethylether (DEE)^8^,^36^ (11) chloroform (CFM) vs. triethylamine (TEA)^8^,^35^ (12) 1,2- propanediamine (12PDA) vs. propylene glycol (PG); (13) 1,2- propanediamine (12PDA) vs. 1,3- propanediol (13PDO)^23^,^32^. The correlation for the systems of (3), (4) and (5) is given in the insert.

The results in this work allow us to check the presumably quantitative correlation between *T_g_* and Δ*H_mix_* in binary mixtures. In addition to the experimental measurements reported here, more *T_g_*^8^,^21^,^25^,^29^,^32^ and Δ*H_mix_* values^21^,^22^,^23^,^33^,^34^,^35^,^36^,^37^ are collected. Fig. 6 shows a plot between Δ*T_g_*/*T_g_* and Δ*H_mix_* measured at 298 K for 13 systems with Δ*H_mix_* ranging from 1044 J/mol to −3900 J/mol. Strikingly, a nearly linear correlation is revealed, and positive Δ*T_g_*/*T_g_* values of mixtures are found to be enlarged with increasingly negative Δ*H_mix_*. Note that although systems with positive Δ*H_mix_* are relatively fewer, the available data in the three systems basically follow the correlation showing that increasingly positive Δ*H_mix_* corresponds to enhanced negative *T_g_* deviation (Δ*T_g_*/*T_g_*). Note that in Fig. 6 there are a few systems that somehow deviate from the master line such as methanol vs. triethylamine^29^,^33^. This may arise partly from the error in Δ*H_mix_* values, since several different Δ*H_mix_* values can be found for a mixing system^38^. However, the minor errors do not affect the demonstration of the explicit relationship between the thermodynamics and the glass transition behaviors in binary mixtures. Compared with the results in Figs. 3 and 4 expressed by the CK and GT models, Fig. 6 consequently implies that Δ*H_mix_* could be a more decisive factor in affecting the *T_g_* behaviors in mixtures.

Fig. 6 includes a typical glass forming binary system of 2-ethyl-1-hexanol (2E1H) and 2-ethyl-1-hexylamine (2EHA), and the accurate *T_g_* data show a large and positive deviation with Δ*T_g_*/*T_g_* reaching 5.6%^39^. The enthalpy of mixing in the system is not available. Yet, the value could be roughly evaluated from the mixtures composed of the homologous liquids with similar molecular structures and chemistry. For example, the Δ*H_mix_* maximum at 298 K reaches −2905 J/mol for the system of butyl amine vs. 1-butanol^26^, which is comparable with the value of −2755 J/mol for the system of amylamine vs. 1-butanol^27^. It is seen that the absolute values of the negative Δ*H_mix_* for mixtures of monoalcohols and monoamines decrease with the carbon number in the backbones of either alcohols or amines, and also decrease with the enhanced steric hindrance due to the introduction of branched chains^26^,^27^,^28^,^40^. Approximately, the Δ*H_mix_* maximum for the mixtures of 2E1H vs. 2EHA is evaluated to be ~−2200 J/mol. The values of Δ*H_mix_* and Δ*T_g_*/*T_g_* (5.6%^39^) for the 2E1H - 2EHA mixtures appear to coincide well with the correlation shown in Fig. 6.

The mixing systems of positive Δ*H_mix_* are much less available. The system of 1-butanol vs. 1-bromobutane is reported to have a quite large and positive Δ*H_mix_* with the maximum reaching 1044 J/mol at 298 K^41^. The composition dependence of the kinetic glass transition temperature, *T_g-kin_*, defined by the temperature where the *α*-relaxation time approaches 100 s^39^,^42^, can be obtained based on the studies of the dielectric relaxation dynamics for the pure components and the mixtures investigated by Goresy *et al*^43^ and by Murthy *et al*^44^. When combining the data from the two groups, a negative *T_g_* deviation with the maximum of Δ*T_g_*/*T_g_* to be −3.2% is yielded. The result is comparable with the predicted one by the correlation in Fig. 6.

For ideally mixing systems with Δ*H_mix_* = 0, a common observation is the negative *T_g_* deviation from the ideal mixing rule, as shown in Figs. 5 and 6 for the MMT vs. MOT mixtures. This is the typical feature driven by mixing entropy^29^,^45^. According to the Adam-Gibbs' configurational entropy (*S_c_*) theory^46^, *τ*(*T*) = *τ*_0_ exp(Δ*μC*_AG_/*TS_c_*), where *τ*_0_ is the pre-exponent having the phonon time, Δ*μ* is potential barrier hindering molecular rearrangement, and *C_AG_* is a constant^47^,^48^,^49^, if the intermolecular interaction keeps unchanged (Δ*H_mix_* = 0), the variation in Δ*μ* is negligible, and the increase in *S_c_* during mixing at a fixed temperature would correspond to a decrease in *τ*(*T*), which makes the relaxation time curves move toward low temperature regions in activation plots (log *τ*(*T*) vs. 1/*T*), and finally leads to a decrease in *T_g-kin_* (or *T_τ = 100 s_*). Furthermore, for the small and comparably-valued Δ*H_mix_* of opposite sign, the case with positive Δ*H_mix_* (for example, the *o*- vs. *m*- MMB system in the inset of Fig. 6) would correspond to relatively larger *T_g_* shift (negative) than the shift (positive) in the case with negative Δ*H_mix_* (for example, the *o*- vs. *m*- DBB system).

In addition to the correlation between Δ*H_mix_* and the *T_g_* shifts in glass forming mixtures, recent studies explored the liquid fragility in various mixtures with varying Δ*H_mix_*, which defines how rapidly the liquid dynamic properties (viscosity or relaxation time) change with temperature at *T_g_*, and found a general trend of negative deviations from the ideal mixing rule^25^, somehow regardless of the enthalpy of mixing. Combining the impacts of Δ*H_mix_* on *T_g_* and fragility in mixtures, it is evident that large and negative enthalpy of mixing would correspond to the high viscosity within supercooled liquid regions^50^, which further favors kinetically glass formation. This rationalizes the empirical criterion that the large and negative enthalpy of mixing is a crucial consideration in order to prepare multicomponent bulk metallic glasses^51^,^52^.

Finally, the present results of the quantitative correlation between Δ*H_mix_* and the composition dependence of *T_g_* in mixtures can provide a guidance to estimating the *T_g_* for materials which are extremely difficult to be vitrified, such as benzene. For the mixtures containing benzene, although a complete dataset of both mixing thermodynamics and glass transition are not available, considering the fact that for most mixtures the composition dependences of *T_g_* generally show a similar trend as the composition dependence of viscosity^29^,^53^, the latter can consequently be used to roughly estimate the former. Δ*H_mix_* and viscosity for benzene vs. decane mixtures have been reported, showing a positive Δ*H_mix_*^54^ but negative deviation in the composition dependence of viscosity^55^, which could imply the negative deviation in the composition dependence of *T_g_*. This agrees with the observation in the present work for the correlation between the mixing thermodynamics and glass transitions in various mixtures. It is therefore speculated that, provided that the enthalpy of mixing is given for the glass forming miscible mixtures containing benzene, the reliable extrapolation towards the *T_g_* of pure benzene can be ensured based on the accessible *T_g_* values of the mixtures. Such endeavor will be conducted in further study.

## Methods

### Chemicals

*o*- and *m*- isomeric systems of methoxymethylbenzene (MMB) and dibromobenzene (DBB) were selected in this study with the former system having small and positive Δ*H_mix_* and the latter system small and negative Δ*H_mix_*^22^. The system of 1, 2- propanediamine (12PDA) vs. propylene glycol (PG) was selected for the considerably large and negative Δ*H_mix_* generated during mixing^23^. For comparison, the results for the system of benzil (BZL) vs. *m*-nitroaniline (MNA) featured by the remarkable large and positive Δ*H_mix_* in our recent studies were used^21^. *o-* MMB (Alfa Aesar 99%), *m-* MMB (Alfa Aesar 99%), *o-* DBB (Alfa Aesar 98%), *m-* DBB (Alfa Aesar 97+%), 12PDA (Sigma Aldrich 99%), PG (Sigma Aldrich 99%) were used as received.

### Thermodynamic measurements

A Perkin-Elmer differential scanning calorimeter (Diamond DSC) was used for the calorimetric determination of the heat capacity curves, and the glass transition temperatures, *T_g_*. A sapphire sample (31.4 mg) was used for the *C_p_* standard. The sample was initially quenched to low temperatures at the accessible highest cooling rate in the calorimeter to guarantee the complete vitrification, and a subsequent heating-cooling-reheating cycle was performed around the glass transition at a fixed rate of 20 K/min. The calorimetric quantities for the systems are determined from the reheating *C_p_* curves. The sapphire and baseline heat curves were obtained following the same procedure. The DSC was calibrated using indium and cyclohexane prior to the measurements^56^.

## Author Contributions

W.K.T., Y.X.W., P.Z. and L.-M.W. finished the experiments and wrote the main manuscript. X.L., S.H.J. and Y.J.T. provide experimental and writing guidance. All authors reviewed the manuscript.
